# Primary Gingival Mucosal Melanoma: A Case Report and Comparison With Cutaneous Melanoma

**DOI:** 10.7759/cureus.112748

**Published:** 2026-07-15

**Authors:** Juan Berríos Cordero, Arnaldo Figueroa, Paula Ruiz, Lisa M Torres Colón, Pablo Mojica-Mañosa

**Affiliations:** 1 General Surgery, School of Medicine, Medical Sciences Campus, University of Puerto Rico, San Juan, PRI; 2 School of Medicine, Universidad Iberoamericana (UNIBE), Santo Domingo, DOM

**Keywords:** cutaneous malignant melanoma, head and neck melanoma, mucosal malignant melanoma, neck dissection, oral melanoma, surgical case reports

## Abstract

Mucosal melanoma (MM) is a rare and highly aggressive malignancy arising from melanocytes located within mucosal surfaces. Unlike cutaneous melanoma (CM), MM develops in non-sun-exposed mucosa, frequently harbors KIT or SF3B1 mutations, demonstrates a lower tumor mutational burden, and exhibits reduced responsiveness to immunotherapy. These biological differences distinguish MM from CM and contribute to important differences in staging, prognosis, and management.

We describe the case of a 71-year-old male who presented with a newly identified pigmented lesion involving the right inferior gingival mucosa. His medical history included type 2 diabetes mellitus, dyslipidemia, and essential hypertension. Biopsy confirmed malignant MM. Positron emission tomography/computed tomography (PET/CT) demonstrated a 1.3 × 1.2 cm fluorodeoxyglucose-avid lesion (SUV max 9.6) without evidence of mandibular invasion, cervical nodal disease, or distant metastasis. The patient underwent radical excision of the lesion with right selective neck dissection (levels I-III). Histopathologic examination confirmed MM (pT3N1) with positive deep margins and metastatic involvement of two regional lymph nodes.

This case highlights the diagnostic and therapeutic challenges of oral MM and emphasizes the importance of recognizing the biological and clinical differences between MM and CM. Routine, thorough intraoral soft-tissue examination and early biopsy of suspicious pigmented oral lesions are essential to facilitate timely diagnosis and management of this aggressive malignancy.

## Introduction

Mucosal melanoma (MM), which accounts for approximately 1%-2% of all melanoma cases, is a rare and aggressive malignancy that arises from melanocytes located within mucosal surfaces [1,2]. Unlike the far more common cutaneous melanoma (CM), oral MM develops independently of ultraviolet (UV) radiation and carries a substantially poorer prognosis. In the oral cavity, MM most commonly involves the maxillary gingiva and hard palate, whereas mandibular lesions, as seen in the present case, are comparatively uncommon [3]. Because oral MM frequently remains asymptomatic during its early stages, diagnosis is often delayed until advanced disease is present, contributing to poorer clinical outcomes [2,4].

Oral MM is particularly challenging to diagnose because early lesions may resemble benign pigmented oral lesions, melanosis, amalgam tattoos, or vascular lesions [3,4]. Common presenting symptoms include oral pigmentation, ulceration, bleeding, pain, loosening of teeth, or mass effect; however, many lesions remain asymptomatic during early growth, contributing to delayed diagnosis and advanced-stage presentation [2-4]. Unlike CM, which benefits from routine skin surveillance and earlier visual detection, oral MM is frequently identified incidentally during dental or oral examinations [3,4].

MM differs fundamentally from CM in its underlying biology. MM develops in mucosa not exposed to UV radiation, exhibits a lower tumor mutational burden, and is enriched for mutations involving KIT, SF3B1, NF1, and SPRED1 [5]. In contrast, CM is strongly associated with UV exposure and commonly harbors mutations in BRAF or NRAS [1,5]. These molecular differences contribute to distinct clinical behavior, therapeutic responses, and overall prognosis, with MM generally demonstrating poorer outcomes and reduced responsiveness to systemic therapies [5,6].

Staging also differs substantially between MM and CM. According to the AJCC 8th edition staging system, all primary head and neck MMs are classified as T3 or higher at diagnosis, reflecting the aggressive nature of this disease [7]. In contrast, CM staging is determined by Breslow thickness, ulceration status, and sentinel lymph node involvement [7]. Furthermore, while sentinel lymph node biopsy plays an established role in the staging and management of CM, its prognostic utility in MM remains less clearly defined [8].

The differential diagnosis of pigmented oral lesions is broad and includes benign and malignant entities such as oral melanotic macules, melanocytic nevi, amalgam tattoos, physiologic pigmentation, drug-induced hyperpigmentation, Kaposi sarcoma, and metastatic melanoma. Because early oral MM may closely resemble these lesions, histopathologic examination remains essential for establishing a definitive diagnosis.

We present a case of primary gingival MM and provide a comparative review of MM and CM, highlighting important differences in epidemiology, molecular biology, staging, prognosis, and management.

## Case presentation

A 71-year-old male with a history of type 2 diabetes mellitus, essential hypertension, and dyslipidemia, and a lifelong never-smoker, presented in June 2025 with a newly identified pigmented lesion involving the right inferior gingival mucosa. The patient denied pain, bleeding, dysphagia, weight loss, or obstructive symptoms. The lesion was initially evaluated by his dentist, who referred him to a maxillofacial surgeon for further assessment. Incisional biopsy confirmed malignant MM, prompting referral to medical oncology and subsequently surgical oncology for definitive management.

Physical examination demonstrated an exophytic, firm lesion involving the right inferior gingival mucosa adjacent to the first molar. The lesion was mobile, non-bleeding, and not fixed to the underlying mandible. No cervical lymphadenopathy was appreciated clinically. Baseline laboratory evaluation demonstrated no significant abnormalities suggestive of an infectious, hematologic, or metabolic etiology. Relevant laboratory findings are summarized in Table 1.

**Table 1:** Baseline laboratory evaluation at initial presentation. Laboratory studies obtained during the initial diagnostic evaluation, including hematologic and biochemical parameters with corresponding reference ranges.

Laboratory test	Value	Reference range
WBC	14.98 ×10³/µL	4.0-11.0 ×10³/µL
Hemoglobin	15.0 g/dL	13.5-17.5 g/dL
Platelets	241 ×10³/µL	150-400 ×10³/µL
Glucose	231 mg/dL	70-99 mg/dL
Creatinine	1.20 mg/dL	0.7-1.3 mg/dL
Sodium	140 mmol/L	135-145 mmol/L
Potassium	4.21 mmol/L	3.5-5.0 mmol/L
Calcium	9.90 mg/dL	8.5-10.5 mg/dL

**Figure 1:**
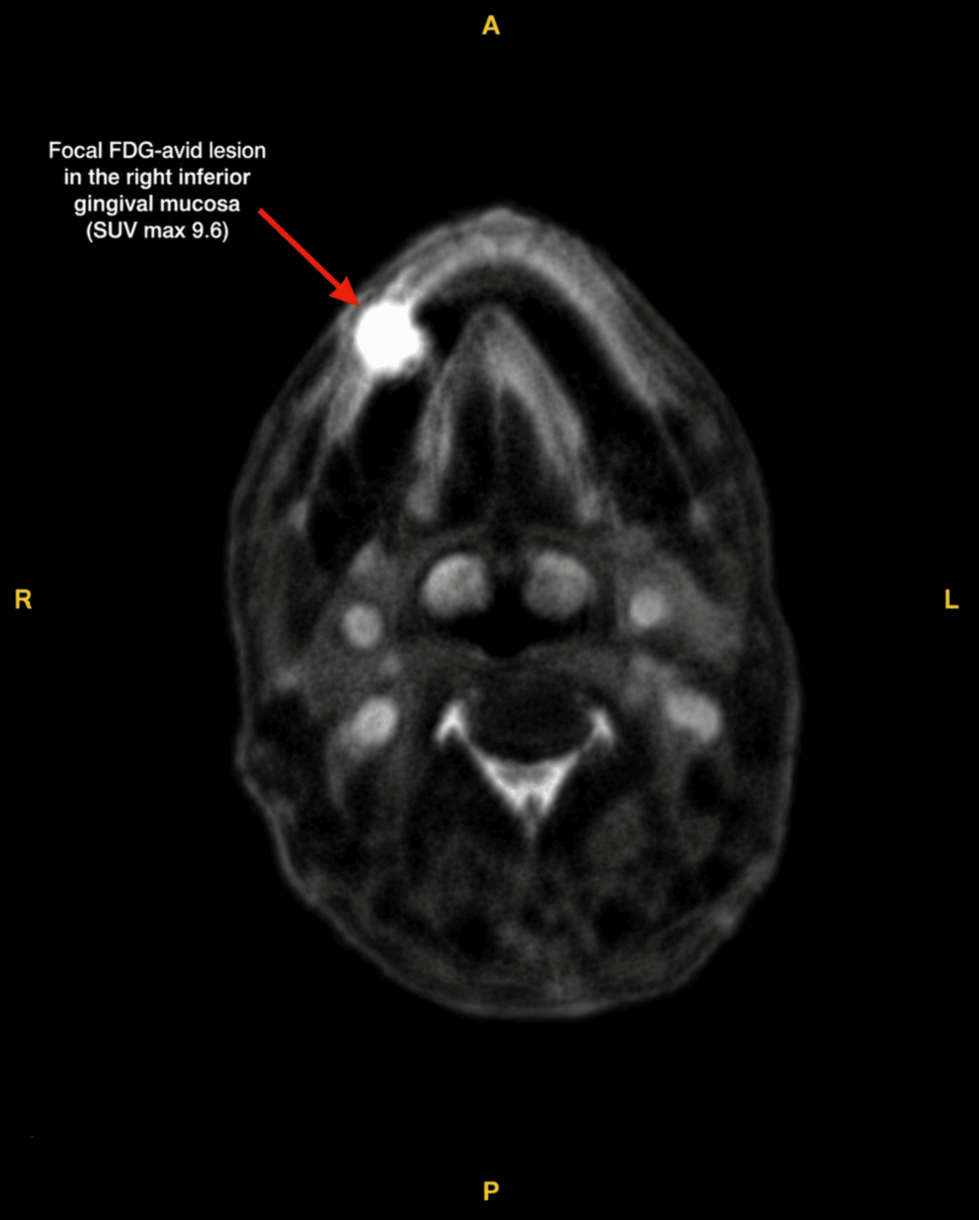
Positron emission tomography/computed tomography (PET/CT) showing a fluorodeoxyglucose (FDG)-avid primary gingival mucosal melanoma as a focal lesion involving the right inferior gingival mucosa (SUVmax, 9.6, red arrow).

PET/CT performed on July 21, 2025, demonstrated a fluorodeoxyglucose (FDG)-avid lesion (SUV_max_ 9.6) measuring 1.3 cm × 1.2 cm without gross mandibular invasion, cervical nodal disease, or distant metastasis (Figure 1).

**Figure 2:**
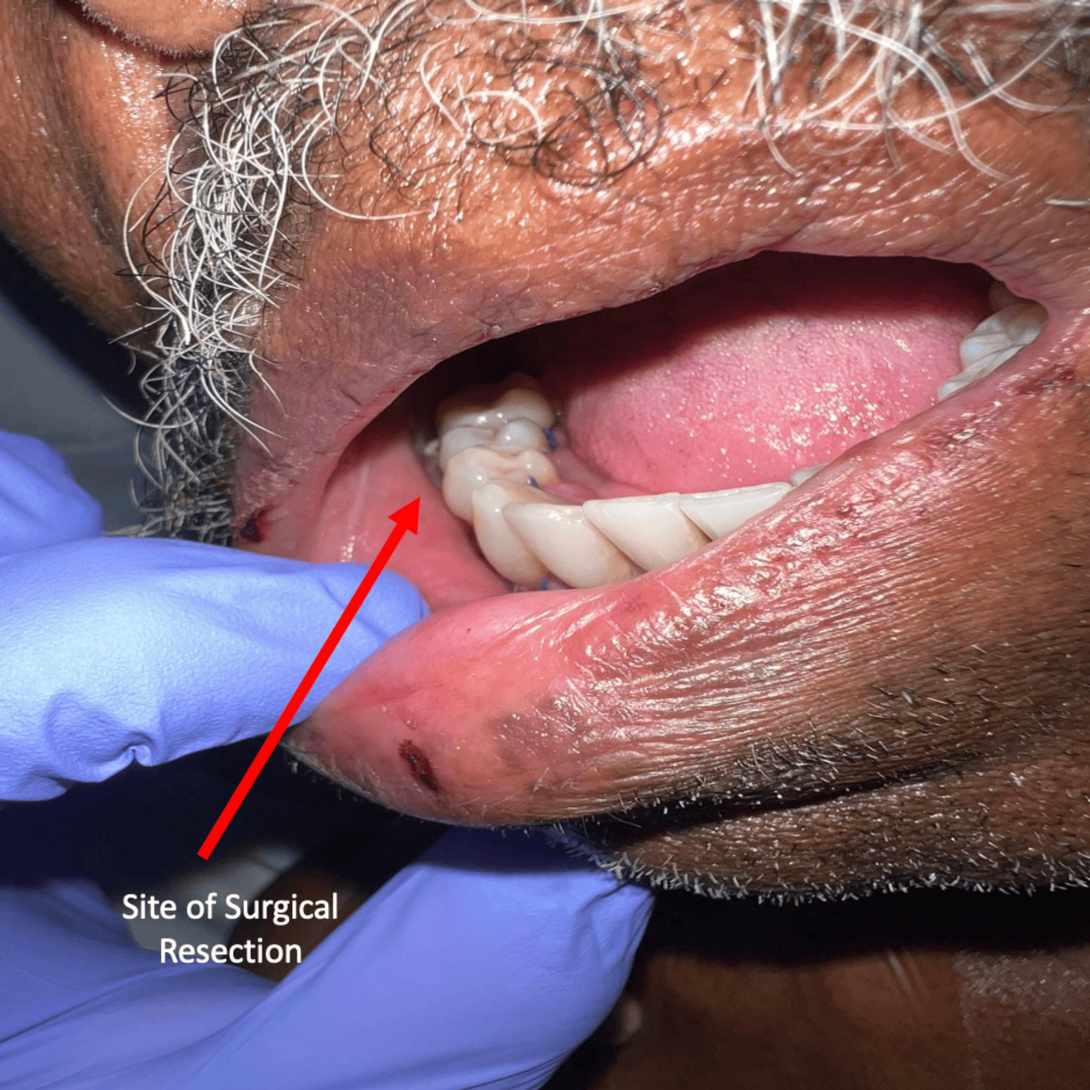
Postoperative appearance following radical excision of gingival mucosal melanoma. Postoperative intraoral photograph showing the surgical site following radical excision of a mucosal melanoma involving the right inferior gingival mucosa. The arrow indicates the tumor resection site adjacent to the mandibular first molar. The patient recovered without immediate postoperative complications.

The patient subsequently underwent radical excision of the gingival melanoma with right selective neck dissection (levels I-III) and primary closure. Intraoperatively, the lesion appeared localized to the right inferior gingival mucosa without gross mandibular invasion. Estimated blood loss was approximately 300 mL. The patient tolerated the procedure well, was extubated in stable condition, and recovered without postoperative complications. Postoperative appearance of the surgical site is shown in Figure 2.

**Figure 3:**
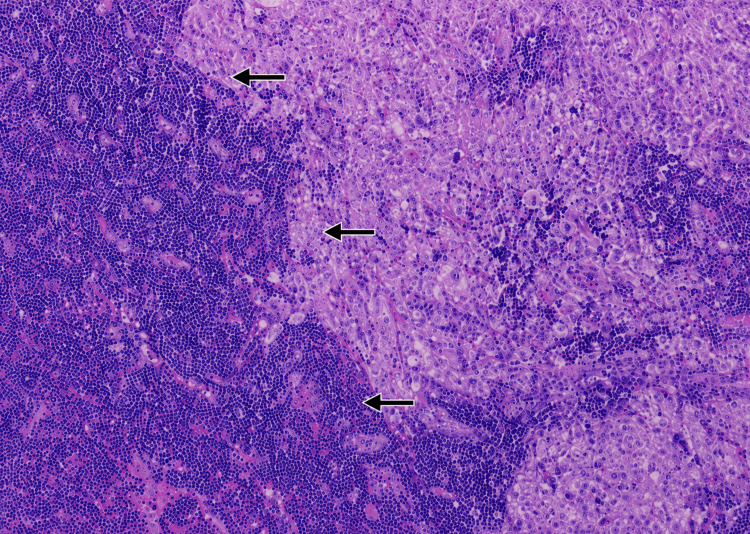
Low-power histopathologic examination of gingival mucosal melanoma. Low-power hematoxylin and eosin (H&E) photomicrograph (100×) demonstrating infiltrative growth of atypical melanocytic cells within the gingival mucosa. Arrows indicate areas of tumor infiltration.

**Figure 4:**
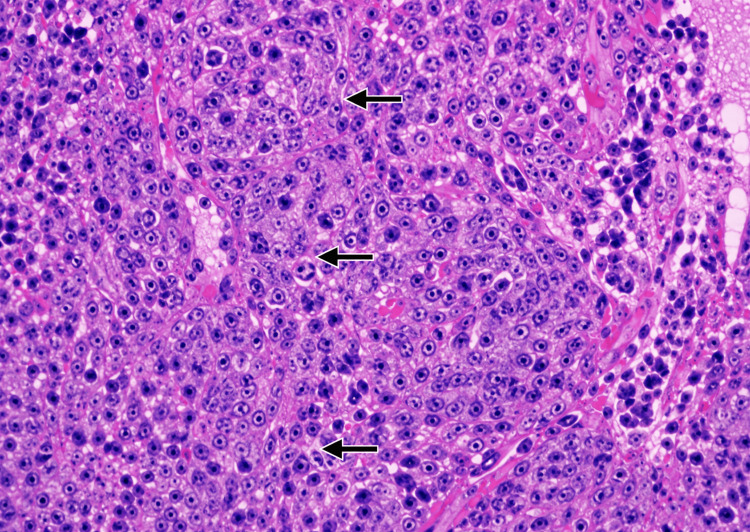
High-power histopathologic examination demonstrating cytologic atypia. High-power hematoxylin and eosin (H&E) photomicrograph (400×) demonstrating pleomorphic melanocytic cells with hyperchromatic nuclei and prominent nucleoli. Arrows indicate representative atypical melanocytes.

**Figure 5:**
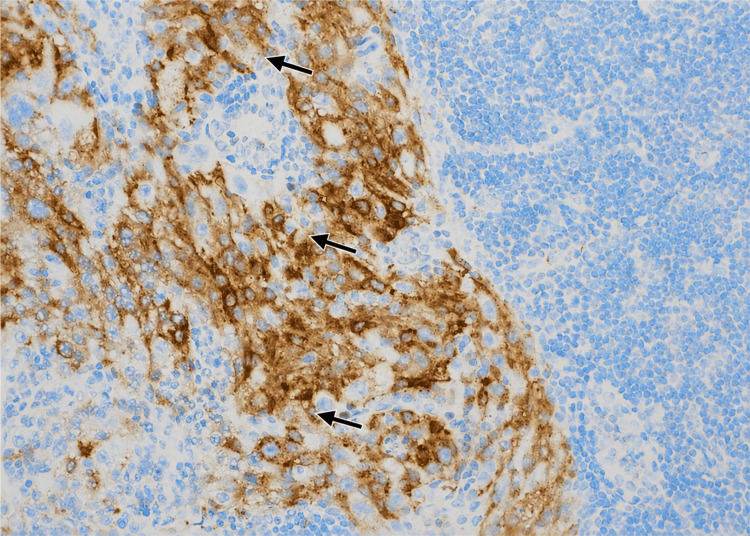
Melan-A immunohistochemical staining of mucosal melanoma. Melan-A immunohistochemical staining (400×) showing diffuse cytoplasmic positivity in tumor cells, supporting melanocytic differentiation. Arrows indicate representative Melan-A-positive cells.

**Figure 6:**
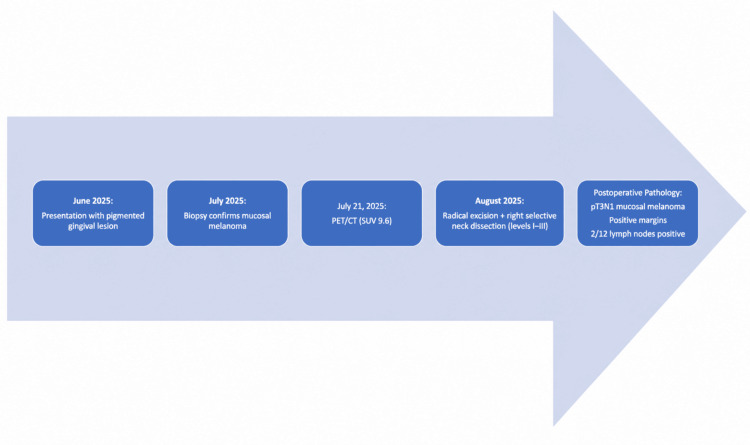
Clinical timeline of diagnosis and management. Timeline summarizing the patient's clinical course from initial presentation through diagnostic evaluation, surgical management, and final pathologic staging. Created by the authors using Microsoft PowerPoint (Microsoft Corporation, Redmond, WA). PET/CT, positron emission tomography/computed tomography

Histopathologic evaluation demonstrated a 1.2-cm unifocal MM with positive deep margins. Low-power examination revealed an infiltrative proliferation of atypical melanocytic cells involving the mucosal stroma with loss of normal tissue architecture (Figure 3). High-power microscopy demonstrated marked cytologic atypia characterized by pleomorphic melanocytic cells with hyperchromatic nuclei and prominent nucleoli (Figure 4). Immunohistochemical staining demonstrated diffuse positivity for Melan-A and SOX-10, confirming melanocytic differentiation (Figure 5). No lymphovascular or perineural invasion was identified. Two of 12 lymph nodes harvested during neck dissection demonstrated metastatic melanoma, with the largest metastatic focus measuring 0.5 cm. According to the AJCC 8th edition staging system for head and neck MM, the final pathologic stage was pT3N1 [7]. A summary of the patient's clinical course is provided in Figure 6.

## Discussion

The present case highlights the aggressive clinical behavior of oral MM and the unique challenges associated with its diagnosis and management. Despite the absence of distant metastatic disease at presentation, our patient demonstrated nodal involvement and positive surgical margins, both of which are recognized adverse prognostic factors associated with increased risk of recurrence and decreased survival [2,3].

Epidemiology and presentation

CM is the most common form of melanoma and is strongly associated with ultraviolet radiation exposure. In contrast, MM represents approximately 1%-2% of all melanomas and arises from melanocytes located within mucosal surfaces [1,2]. Oral MM most frequently involves the maxillary gingiva and hard palate and often presents asymptomatically or with nonspecific symptoms, contributing to delayed diagnosis and advanced-stage disease at presentation [2-4]. Early oral MM may initially present as a small, asymptomatic flat pigmented macule or patch with subtle color variation before progressing to a nodular, ulcerated, or exophytic lesion [2-4]. Because these early clinical findings are often overlooked or mistaken for benign pigmented lesions, diagnosis is frequently delayed until more advanced disease develops. Key differences between MM and CM are summarized in Table 2.

**Table 2:** Key differences between mucosal melanoma and cutaneous melanoma. Sources: [1,4-6,9-16].

Characteristic	Mucosal melanoma (MM)	Cutaneous melanoma (CM)
Incidence	Rare; approximately 1%-2% of all melanomas	Most common melanoma subtype
Primary site	Mucosal surfaces (oral cavity, sinonasal tract, anorectal region, vulvovaginal tract)	Sun-exposed skin
Association with UV exposure	No established association	Strongly associated with ultraviolet radiation
Typical age at presentation	Sixth to seventh decade of life	Variable, often younger than MM
Common clinical presentation	Pigmented or amelanotic mucosal lesion, ulceration, bleeding, mass effect	Pigmented cutaneous lesion with ABCDE characteristics
Diagnostic delay	Common due to occult location and nonspecific symptoms	Less common due to visible skin lesions
Tumor mutational burden	Lower	Higher
Common genetic alterations	KIT, SF3B1, NF1, SPRED1	BRAF, NRAS, NF1
BRAF mutation frequency	Uncommon	Common
Immunogenicity	Lower	Higher
Response to immune checkpoint inhibitors	Reduced response rates	Higher response rates
Role of sentinel lymph node biopsy	Limited and less clearly defined	Standard component of staging
AJCC T classification (head and neck)	All tumors staged as T3 or higher	Based on the Breslow thickness and ulceration
Role of adjuvant radiotherapy	Frequently used for locoregional control	Less commonly required
Five-year survival	Approximately 20%-40%	Frequently exceeds 80%-90%, depending on the stage
Overall prognosis	Poor	Generally more favorable

Biology

Important molecular differences distinguish MM from CM. MM commonly harbors mutations involving KIT, SF3B1, NF1, and SPRED1, whereas BRAF mutations are comparatively uncommon [5,6]. As a result, targeted therapies commonly used in CM are often less applicable in MM. The reduced responsiveness of MM to immunotherapy is believed to be associated with lower tumor mutational burden and a comparatively less inflamed immune microenvironment [9,10]. In contrast to CM, MM demonstrates reduced ultraviolet-induced mutagenesis and lower neoantigen formation, potentially contributing to decreased immunogenicity and diminished response to immune checkpoint inhibition [5,9,10]. CM generally exhibits greater responsiveness to immunotherapy due to its higher tumor mutational burden and increased prevalence of actionable driver mutations [1,5].

Staging

The staging systems for MM and CM differ substantially. CM staging relies heavily on Breslow thickness, ulceration status, and regional lymph node involvement [7]. Unlike CM, Breslow thickness is not routinely applied to oral MM because the oral mucosa lacks a histologic equivalent of the papillary and reticular dermis, limiting its prognostic utility. In contrast, all primary head and neck MMs are staged as T3 or higher under the AJCC 8th edition because of their aggressive biological behavior and poor prognosis [7]. This distinction reflects the unique clinical course of MM and underscores the limitations of directly applying CM staging principles to mucosal disease.

Surgery and regional control

Complete surgical excision remains the cornerstone of treatment for both MM and CM. However, achieving adequate surgical margins is frequently more difficult in MM because of anatomic constraints and proximity to critical structures [11]. This challenge was demonstrated in our patient, whose final pathology revealed positive deep margins despite radical excision. While sentinel lymph node biopsy is routinely incorporated into the management of CM, its role in MM remains less clearly established and continues to be an area of ongoing investigation [8,12].

Adjuvant radiotherapy

Adjuvant radiotherapy is commonly employed in MM to improve locoregional disease control, particularly in patients with positive margins, locally advanced disease, or regional nodal involvement [13,14]. Although current evidence suggests improvement primarily in locoregional control rather than overall survival, radiotherapy remains an important component of multimodal treatment strategies for selected patients [13,14]. This approach is used less frequently in CM, where local control is often achieved through surgery alone.

Systemic therapy

Immune checkpoint inhibitors have transformed the management of advanced melanoma; however, outcomes differ between MM and CM. Patients with CM generally experience higher response rates to PD-1-based therapies, whereas MM demonstrates comparatively lower responsiveness [15,16]. Combination therapy with nivolumab and ipilimumab has shown improved outcomes compared with PD-1 monotherapy in MM and remains an important treatment option for advanced disease [15]. Despite lower response rates relative to CM, immune checkpoint inhibitors continue to play a critical role in the management of unresectable or metastatic MM. Additionally, patients with KIT-mutated tumors may benefit from targeted therapies such as imatinib [6].

Prognosis

MM carries a substantially worse prognosis than CM, with reported five-year survival rates ranging from approximately 20% to 40% [2,3]. Several factors contribute to these poorer outcomes, including delayed diagnosis, anatomically constrained surgical margins, early hematogenous dissemination, and reduced responsiveness to currently available systemic therapies [2,3,10]. Positive surgical margins and nodal metastases, both present in our patient, are among the strongest predictors of recurrence and diminished survival [2,3]. Furthermore, emerging evidence suggests that differences in the tumor immune microenvironment may contribute to the inferior therapeutic responses observed in MM compared with CM [9,10]. As a single case report, our findings should be interpreted within the context of the existing literature and are not intended to establish definitive management recommendations. Further studies are warranted to better define optimal diagnostic and therapeutic strategies for oral MM.

## Conclusions

Primary oral MM remains a rare but highly aggressive malignancy with a substantially poorer prognosis than CM because of delayed diagnosis, distinct molecular characteristics, and limited therapeutic responsiveness. This case highlights the importance of recognizing the biological and clinical differences between MM and CM when considering diagnosis, staging, and management.

For dental professionals, oral surgeons, and general practitioners, this case underscores the importance of performing routine, thorough intraoral soft-tissue examinations and maintaining a high index of suspicion for persistent or unexplained pigmented oral lesions. Early recognition, prompt biopsy, and timely referral to appropriate specialists are critical to facilitating earlier diagnosis and may improve clinical outcomes in this aggressive disease. As a single case report, these findings should be interpreted within the context of the existing literature, and further studies are warranted to better define the optimal diagnostic and therapeutic approaches for oral MM.
